# Glycemic Control in Kenyan Children and Adolescents with Type 1 Diabetes Mellitus

**DOI:** 10.1155/2015/761759

**Published:** 2015-10-01

**Authors:** Thomas Ngwiri, Fred Were, Barbara Predieri, Paul Ngugi, Lorenzo Iughetti

**Affiliations:** ^1^Pediatric Endocrinology Training Center, Gertrude's Hospital, Nairobi, Kenya; ^2^Pediatrics Clinic, Kenyatta National Hospital, Nairobi, Kenya; ^3^Department of Pediatrics, University of Modena and Reggio Emilia, 41124 Modena, Italy; ^4^Endocrinology Clinic, Kenyatta National Hospital, Nairobi, Kenya

## Abstract

*Background*. Type 1 diabetes mellitus (T1DM) is the most common endocrine disorder in children and adolescents worldwide. While data about prevalence, treatment, and complications are recorded in many countries, few data exist for Sub-Saharan Africa. The aim of this study was to determine the degree of control in patients with T1DM aged 1–19 years over a 6-month period in 3 outpatient Kenyan clinics. It also sought to determine how control was influenced by parameters of patient and treatment. *Methods*. Eighty-two children and adolescents with T1DM were included in the study. Clinical history regarding duration of illness, type and dose of insulin, and recent symptoms of hypoglycemia/hyperglycemia were recorded. Glycaemia, HbA1c, and ketonuria were tested. HbA1c of 8.0% and below was defined as the cut-off for acceptable control. *Results*. The median HbA1c for the study population was 11.1% (range: 6.3–18.8). Overall, only 28% of patients had reasonable glycemic control as defined in this study. 72% therefore had poor control. It was also found that age above 12 years was significantly associated with poor control. *Conclusions*. African children and with T1DM are poorly controlled particularly in adolescents. Our data strongly support the necessity of Kenya children to receive more aggressive management and follow-up.

## 1. Introduction

T1DM is the most common endocrine-metabolic disorder in children and adolescents worldwide, with a prevalence of 190 per 100000 among school aged children in USA and an annual incidence ranging from 1.7 per 100000 (China) [1] to 40 per 100000 (Finland) [2]. Over the last three decades, the incidence of T1DM has been also on the rise worldwide [1–3].

Survey of the published information on T1DM in African populations reveals that most series do contain several children and a significant number of teenagers [4–7]. This shows that T1DM is not rare in African countries contrary to the widely held belief.

With the few data available on Sub-Saharan African children [4], incidence in Tanzania was estimated to be 1.5/100,000 [5], and an increase in incidence in Sudan from 9.5/100,000 in 1991 to 10.3/100,000 in 1995 has been shown [6]. Finally, in Africa, a global incidence of 6.4/100.000/year was reported [7].

Due to limitations in study methods and design, many of these studies do not provide reliable population data; however, International Diabetes Federation (IDF) estimates that there were a total of almost 39,000 prevalent cases of children with type 1 diabetes in Sub-Saharan Africa in 2013 [7].

The prevalence of type 1 diabetes in Kenyan children and adolescents is totally unknown. However, approximately 100 patients below the age of 20 years are being followed up in Nairobi. Despite the apparently small numbers of these patients, diabetes is a major health concern because it is a lifelong disease, which uses up substantial financial resources on both personal and national levels. On the other hand, diabetic children if well managed can enjoy reasonable well-being and personal independence [8].

The main long-term complications of T1DM result from the effects of prolonged high blood glucose. The Diabetes Control and Complications Trial (DCCT) showed conclusively that the control of blood glucose has a direct and significant effect on the development of the triad (nephropathy, retinopathy, and neuropathy) of long-term complications of T1DM. A reduction of 76% in the risk for development of retinopathy when average blood glucose and HbA1c are maintained at 8.6 mmol/L and 7.2%, respectively, over a seven-year period (intensively treated group) was demonstrated [9]. The DCCT showed that it is indeed possible to achieve adequate control to make a difference in occurrence of long-term complications. The patient must be well motivated and the healthcare system must be able to give the necessary support in terms of medications and supplies, clinical care, and advice as required. Although euglycemia was not achieved even in the DCCT, the improved control resulted in significant reductions in long-term complications. The control of blood sugar among Kenyan children and adolescents with T1DM has not been reliably evaluated. In the absence of such data, the effectiveness of therapy or any other interventions both currently and in the future cannot be ascertained.

The aim of this study was to determine the degree of glycemic control in children and adolescents with T1DM and to try correlating it with the availability of insulin, its type and dosage, and the role of patient characteristics such as age and duration of illness. This study also aimed at providing a baseline on which future work on T1DM in Kenya can build.

## 2. Methods

The study was carried out at the Kenyatta National Hospital (KNH) diabetic clinic, the Presbyterian Church of East Africa (P.C.E.A.) Kikuyu Hospital, and the diabetes care and training centre in Nairobi in a period of 6 months and submitted in partial fulfillment of the requirements for the degree of Master of Medicine in Pediatrics and Child Health at the University of Nairobi by the first author (Thomas Ngwiri).

Even if these clinics are not specifically designed for children and the most part of patients are adults with T2DM, children with T1DM receive appropriate treatment and education; their diabetes is monitored; and where necessary and possible they are referred to available specialists and support services. The clinics aim to educate and inform patients and their relatives to enable them to achieve best possible control of their condition in a setting with scarce resources. Generally, any patient is periodically reviewed for blood sugar, blood pressure, and body weight measurements. Home blood sugar monitoring is taught to patients who can afford the strips. Currently, none of patients can afford regular HbA1c assays.

All children and adolescents previously diagnosed with T1DM, being managed with insulin at the 3 facilities and with at least 2 clinical assessments in this period, were eligible to participate.

Signed informed consent was obtained from the parent/guardian or subjects below 18 years or from the patient himself if above 18 years of age. Authority was obtained from the local ethical committees.

Medical history of the patient in the preceding three months was taken and recorded on the basis of a structured anamnestic questionnaire with regard to hyperglycemia and/or severe hypoglycemia. Considering the fact that only few patients had glucose monitoring at home, history of polydipsia, polyuria, and nocturia was used as clinical evidence of hyperglycemia. Moreover, on the same basis, history of loss of consciousness or convulsions, dizziness, unusual sweating, or tremors requiring help from a third party and corrected by glucose administration were recorded as severe hypoglycemia.

A history of the insulin dosage, availability, and storage in the last 3 months was also noted. The patient was also being tested on his/her ability to measure the prescribed dose of insulin and these findings compared with glycemic control.

In all subjects, a sample of urine was collected and tested for ketones; moreover, a venous sample of blood was drawn from a peripheral vein and a fasting blood glucose level and HbA1c level were measured.

Fasting blood glucose was measured using the “HemoCue” blood glucose analyzer. HbA1c was determined only at the second clinical assessment using the HbA1c Tina Quant II kit (Roche Diagnostics GmbH, Mannheim, Germany) at the Chemistry Department of the Nairobi Hospital laboratory.

HbA1c normal range for this assay was 4.8–5.9%.

Urine was collected and tested for ketones using the “Bayer Diagnostics” ketone test strip.

The questionnaire and blood and urine samples were given a matching serial number and entered into the study for analysis. The patient's level of control and its implications were explained to the patient/guardian by the attending clinician at the next clinic visit. Patients were said to have reasonable control if they had HbA1c of 8% or less and were said to have poor control if HbA1c was higher than 8%.

The information obtained in this study was discussed with the patient/primary caregivers and the healthcare providers to enhance the quality of care to the study subjects.

All data were then analyzed using the Statistical Package for Social Sciences (SPSS version 11.0) using comparison of means or chi-square as appropriate. Results are shown as mean SD.

Odds ratios were also calculated.

## 3. Results

The entire population of eligible patients visiting the clinics in the 6-month period was recruited. A total of 82 patients (39 males and 43 females, age range: 3–19 years, height: −0.31 ± 0.82 SDS, weight: −0.23 ± 0.53 SDS) were studied. This group represents near half of the registered patients in these clinics and the total of the patients in regular follow-up.

Thirty-nine of the 82 patients (48%) were residents of Nairobi, while the remaining 43 (52%) resided in other districts of Kenya and had to travel to the 3 facilities for treatment.

The mean age at diagnosis was 9.9 ± 4.4 years and the median duration of illness was 3.7 years.

Of the patients identified for the study after meeting the inclusion criteria nobody was excluded from analysis.

Although 90% of the patients were recruited at KNH, it was noted that patients had moved freely among the 3 facilities in the 6 months preceding the study so no attempt was made to analyze control separately for each institution.

The male to female ratio of the study population approached one.

**Figure 1:**
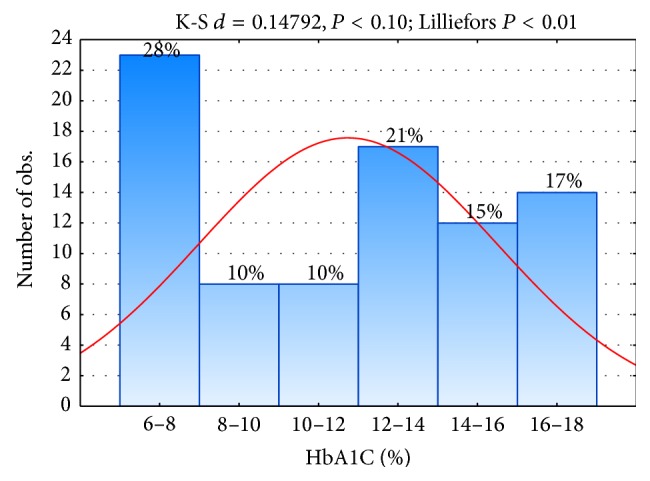
Distribution (%) of HbA1c in children and adolescents.

Values of HbA1c were not normally distributed: their range was 6.4–19%, median HbA1c was 12.1%, and interquartile range was 7.20. The overall prevalence of poor control was 72% when a cut-off HbA1c of 8% was used (Figure 1). Children in the group with lowest levels of HbA1c were in honeymoon period.

The percentage of children experiencing severe hypoglycemia (53.6%) was lower than the percentage of children with hyperglycemia (86%).

Only about one-third of patients in the study had fridges to store their insulin. Control in this group was not significantly better than in those who kept their insulin at room temperature. Those patients using “pots” (improvised refrigerator) also appeared to have similar control to those who stored their insulin at room temperature.

The dose of insulin is highly individualized as it is titrated against the patients control during the course of follow-up. All children received a conventional insulin regimen (mean dose: 0.9 ± 0.4 UI/kg/day; range: 0.5–1.7 UI/kg/day).

In this study, control was compared between those whose insulin dose was at least 0.6 UI/kg and those whose dose fell below this cut-off. Control was similar in the 2 groups.

Fifty-eight of the 82 patients, 69% of patients, used only mixed formulation insulin, while the rest used intermediate acting insulin alone or together with a short acting variety in a home-prepared mixture. In all these patients, this was necessitated by unavailability of mixed formulation and not by preference for an alternative formulation.

Eleven% of patients admitted that they took refined sugars between daily basis and weekly basis as compared to the majority who took them not more than once a month. Control was better in those children who restricted their refined sugar intake, although the difference was not significant.

In this study, only 4% of children >12 years had reasonable control compared to 78% in children <12 years of age.

Statistical analysis demonstrates that patients' age was a determinant of glycemic control (*P* < 0.0001) as confirmed by odds ratio value.

No statistically significant differences were shown for sex, residence, primary care givers, family history of diabetes mellitus, insulin formulation, use of refined sugar, and duration of illness in determining the degree of metabolic control (Tables 1 and 2).

## 4. Discussion

Only 28% of Kenyan children and adolescents with T1DM had reasonable control. This result is partially in agreement with the few studies in African children showing the mean HbA1c above 10.5% [10–13] with a study having the mean HbA1c as high as 12.5% [14]. Our data compare to those of a study from Ethiopia where reasonable control was found in 22% of patients [13] and are better than those obtained in Sudan where reasonable control was found in only 12.5% of patients [10, 11].

As would be expected, control was poorer than that in most patients studied by Hvidøre Study Group [15], with a more prosperous economy and therefore better access to healthcare.

Most likely, the underlying cause is the association between limited insulin supply and near total lack of self-monitoring of blood glucose due to the limited availability of economic resources. The same scarcity of resources blocks the possibility of routinely assessing the level of HbA1c. Therefore, the costs of diabetes result in a formidable barrier to improve the control of this noncommunicable disease. In African countries, the global direct cost of care approaches 300$ corresponding to the annual income of many families. In the absence of a publically funded healthcare system, these costs are borne almost entirely by individuals. In this context, poor disease prognosis with high morbidity and mortality seem to be the unavoidable outcomes [16].

In our population, we showed a high prevalence of both severe hypoglycemia and hyperglycemia. However, these data have to be considered cautiously because they are based on symptoms and not always on glucose measurements. Hyperglycemia, moreover, can be reflecting the shortage of insulin. This can concur with the high recurrence of ketoacidosis in African children with T1DM [17]. Females had slightly poorer glycemic control than their male counterparts as previously shown in other pubertal African girls [14]. Similar findings were observed also in Japan, where the average HbA1c in females remained significantly higher than in males over more than 5 years of follow-up [18]. Adolescent girls experience a significant deterioration in control towards the end of growth [19].

In our study, to live in rural setting does not influence the metabolic control. Thus, the control was similar in all children independently by residing in or out of Nairobi, probably reflecting the relative importance of treatment practice over the physical environment and diverse diets in the successful management of type 1 diabetes. The landmark DCCT [9] had shown that good control was achievable anywhere with aggressive management.

The majority of patients had a parent as the primary care giver. These children did not fare better with their control than those under care of siblings and other relatives. Parents are probably more committed to their children with a chronic illness and ensure better compliance with medication. Children under care of their parents also might enjoy a stable family structure that is more supportive. It has been shown that there was a direct association between parental involvement in insulin administration and metabolic control in diabetic adolescents, although no such association was found with the “wider family functioning” using the Family Assessment Device [20]. Another study [21] also established that control was indeed better in those children whom both parents lived at home. However, the relationship between family functioning (namely, at mealtimes) and children's health outcomes has to be yet fully elucidated [22].

The lack of a difference in our study might be explained by the fact that many Kenyan homes comprise an extended family whose members assume responsibility for the care of a sick sibling or other relative just like a parent would.

Families with experience in the care of another diabetic might be expected to be better suited for the care of a diabetic child by providing appropriate diet and correct and consistent administration of medication and generally offering psychosocial support. The author is not aware of studies that have specifically addressed the quality of care of children with family history of diabetes. In our study population, the control was similar in those with or without diabetic relatives.

The DCCT study [9] had established that control improved with multiple dose injections or continuous subcutaneous infusion of insulin. These recommendations caused a high proportion of children and adolescents with T1DM in developed countries to receive intensive treatment. A crossover study from conventional to flexible multiple daily insulin showed an improvement in both preadolescent control and pubertal children's control [23]. This requires a highly motivated patient who must practice home glucose monitoring several times a day. The economic problems of African countries put an obstacle in the way of intensive treatment. Wide use of mixed formulations recorded in our patients is the standard practice in Africa. As reported by Majaliwa et al. [14], it is unlikely that self-monitoring of blood glucose can be economically sustained by patients living in these countries. It is to underline, however, that all 8 patients with a limited access to self-monitoring are among those with a good metabolic control. Pawar et al. [24], on the other hand, found that the benefits of multiple dose regimens seen in research settings were not always reproducible in routine clinics. They found control was poorer in patients on four as compared to two daily injections in a routine clinic in the UK. In our study, the number of patients on thrice daily injections was extremely small (5% of study subjects). They also had been put on thrice daily injections only after failing to be controlled on twice daily regimen and so were likely to be patients with poor control in the first place.

While in other African countries the storage of insulin can play role in the overall metabolic control [14], this does not happen in our environment where temperatures remain around 25 degrees centigrade. Refrigeration where available should still remain the storage of choice especially in hotter parts of the country where temperatures frequently exceed 25 degrees centigrade. Insulin can still be safely kept at room temperature as long as it is used within one calendar month and the ambient temperature remains below 25 degrees centigrade, a situation that is difficult to guarantee in the tropics. There is no data on the temperatures achieved in “pots.” This should be ascertained and if acceptable the use of “pots” should be promoted more authoritatively as an alternative for patients without access to a refrigerator.

In Kenya and specifically at KNH which has a high turnover of patients on insulin and which must provide the drug at a highly subsidized price (15% of the price at private pharmacy outlets), insulin shortages are not uncommon. The extent and impact of shortages on the glycemic control in Kenya have not been evaluated.

Our study was not designed to verify the report of the patient's compliance and relied on recall. However, only 10 out of 82 patients reported that they had missed their prescribed dose of insulin because they lacked the drug. Those who did so had poorer control than those who reported that they had been compliant throughout the preceding 3 months. While these results are in keeping with the scientific fact that insulin is all-important in control of blood sugar, it is still significant that even those who reported good compliance still had a very poor control. Therefore, we can have some doubts on the real compliance of these patients. On the other hand, our study does not address the difference between the prescribed dose and dispensed dose of insulin and so cannot verify compliance with the prescribed dose.

Elbagir et al. [10] in Sudan had found that patients faced with scarcity of insulin actually reduced their insulin per dose or the number of doses per day in order to stretch their supply.

Tanzanian children with T1DM, in addition to limited supply, reduce spontaneously the prescribed dose of insulin to guaranty a longer period and lower cost of treatment [14]. It is not clear from our study if the same happens in Kenya.

In our patients, age was an important determinant of metabolic control. In agreement, a nationwide study of more than 2500 French children [23] showed age as the most important factor related to control followed by insulin dosage and maternal age. Pawar et al. [24] had similar findings and concluded that mean HbA1c rose with age from 10 years of age over a 6-year follow-up. In the DIABAUD2 study [21], HbA1c was significantly lower in children below 10 years as compared to those between 10 and 15 years. Children diagnosed with diabetes before 12 years of age had slightly poorer control than those diagnosed later and in adolescents the control was unacceptably impaired [25]. Gebre-Yohannes and Rahlenbeck [13] had also found that lower age of onset was a predictor of poor control in Ethiopian children with T1DM. Control was also inversely related to duration of illness. However, the correlation reported in our and other studies between HbA1C and age was not found in Tanzanian children with T1DM [14].

However, it has been shown that there was a delay in the onset of complications of diabetes in those with a longer duration of prepubertal diabetes as compared with the duration of postpubertal disease [23]. This finding has major implications in setting target HbA1c levels for young children who are prone to hypoglycemia.

Our study demonstrated that the prevalence of poor diabetic control in Kenyan children and adolescents is far from being acceptable and the great majority of patients are at high risk for the precocious development of microvascular complications. Moreover, it must be highlighted that severe hypoglycemia is often recorded in African setting and it can complicate the control. As worldwide, Kenyan adolescents with T1DM have particularly poor control so they comprise a special high-risk group in the diabetes clinic. Our data strongly have supported the necessity of children and adolescents with T1DM in Kenya receiving more aggressive management and follow-up and more resources are addressed at the care of this noncommunicable disease.

Just to overcome some structural barriers in coping with diabetes in the African setting [26], in the last years, the assistance to the children with T1DM has improved offering many free blood meters and blood strips and near-free access of insulin, with the help of the Pediatric Endocrinology Training Center, sponsored by the European Society Pediatric Endocrinology and the World Diabetes Foundation.

Moreover, a more strict collaboration has been promoted between adult and pediatric diabetologists and a valuable assistance from the diabetic education unit with nurse and educator has been obtained.

Further studies should be carried out to determine the impact of these increased resources and this new attitude on the metabolic control, especially in adolescent patients.

## Figures and Tables

**Table 1:** Sociodemographic factors and glycemic control.

Factor	HbA1c < 8.0%	HbA1c ≥ 8.0	*χ* ^2^	*P* value	Odds ratio (95% CI)
Sex					
Male	12 (33%)	26 (66%)	0.44	0.623	1.18 (0.53–3.64)
Female	11 (25%)	33 (75%)			
Residence					
Nairobi	13 (33%)	26 (66%)	1.03	0.310	1.65 (0.62–4.36)
Others	10 (23%)	33 (77%)			
Primary caregiver					
Parent	20 (30%)	46 (70%)	0.85	0.356	1.88 (0.48–7.35)
Other	3 (19%)	13 (81%)			
Family history of DM					
Yes	9 (27%)	24 (73%)	0.02	0.897	0.94 (0.35–2.51)
No	14 (29%)	35 (71%)			
Insulin formulation					
Intermediate acting	4 (25%)	12 (75%)	0.195	0.6588	0.75 (0.22–2.64)
Mixed	19 (33%)	43 (67%)			
Use of refined sugar					
Frequent	1 (11%)	8 (89%)	0.6	0.431	0.30 (0.04–2.53)
Rare	21 (30%)	50 (70%)			
Age (current)					
<12 years	21 (78%)	6 (22%)	45.73	<0.0001	92.7 (17.3–496.8)
≥12 years	2 (4%)	53 (96%)			
Age at diagnosis					
<12 years	6 (22%)	21 (78%)	0.32	0.448	0.64 (0.22–1.87)
≥12 years	17 (31%)	38 (69%)			
Duration of illness					
<5 years	19 (31%)	41 (69%)	1.451	0.2285	2.09 (0.62–7.01)
≥5 years	4 (19%)	18 (81%)			

**Table 2:** Insulin handling and glycemic control.

Factor	*N*	Mean HbA1c (standard deviation)	Standard error (SE)	*P* value
Place of storage				
Fridge	28	10.189 (4.1176)	0.7781	0.234
Room temp.	9	12.400 (4.1158)	1.3719	
Place of storage				
Pot	40	11.433 (4.2589)	0.6734	0.539
Room temp.	9	12.400 (4.1158)	1.3719	
Insulin dose				
≥0.6 U/kg/day	54	11.041 (4.1120)	0.5596	0.991
<0.6 U/kg/day	28	11.053 (4.2166)	0.9673	
Dosing regimen				
Twice daily	76	11.030 (4.2464)	0.4936	0.258
Thrice daily	4	12.850 (2.5749)	1.2874	
Accurate measurement of insulin				
Yes	77	10.997 (4.2102)	0.4862	0.573
No	5	13.200 (2.5755)	1.2878	
Missed insulin (unavailability)				
Yes	12	12.756 (4.7101)	1.5700	0.216
No	70	10.910 (4.1020)	0.4938	
